# Association between socioeconomic deprivation and colorectal cancer screening outcomes: Low uptake rates among the most and least deprived people

**DOI:** 10.1371/journal.pone.0179864

**Published:** 2017-06-16

**Authors:** Andrea Buron, Josep M. Auge, Maria Sala, Marta Román, Antoni Castells, Francesc Macià, Mercè Comas, Carolina Guiriguet, Xavier Bessa, Xavier Castells, and the PROCOLON research group

**Affiliations:** 1 Epidemiology and Evaluation Department, Hospital del Mar, Barcelona, Spain; 2 IMIM (Hospital del Mar Medical Research Institute), Barcelona, Spain; 3 REDISSEC (Health Services Research on Chronic Patients Network), Madrid, Spain; 4 Biochemistry and Molecular Genetics Department, Hospital Clínic, Barcelona, Spain; 5 IDIBAPS (August Pi i Sunyer Biomedical Research Institute), Barcelona, Spain; 6 Gastroenterology Department, Hospital Clínic, Barcelona, Spain; 7 University of Barcelona, Barcelona, Spain; 8 CIBERehd (CIBER for Digestive and Liver Diseases), Madrid, Spain; 9 Gòtic Primary Care Center, Catalan Institute of Health, Barcelona, Spain; 10 IDIAP Jordi Gol (Institute in Primary Care Research), Barcelona, Spain; 11 Gastroenterology Department, Hospital del Mar, Barcelona, Spain; 12 Autonomous University of Barcelona, Barcelona, Spain; Baylor University Medical Center, UNITED STATES

## Abstract

**Background:**

Screening with faecal occult blood tests reduces colorectal cancer-related mortality; however, age, sex and socioeconomic factors affect screening outcomes and could lead to unequal mortality benefits. The aim of this study was to describe the main outcomes of the population-based Barcelona colorectal cancer screening programme (BCRCSP) by deprivation.

**Methods:**

Retrospective study of the eligible population of the first round of the BCRCSP. Participants’ postal addresses were linked with the MEDEA database to obtain the deprivation quintiles (Dq). Chi-squared tests were used to compare proportions across variables and logistic regression was used to estimate the adjusted effects of age, sex and deprivation on uptake, FIT positivity, colonoscopy adherence and advanced neoplasia detection rate.

**Results:**

Overall uptake was 44.7%, higher in Dq2, 3 and 4 (OR 1.251, 1.250 and 1.276, respectively) than in the least deprived quintile (Dq 1), and lowest in Dq5 (OR 0.84). Faecal immunochemical test (FIT) positivity and the percentage of people with detectable faecal haemoglobin below the positivity threshold increased with deprivation. The advanced neoplasia detection rate was highest in Dq4.

**Conclusion:**

Unlike most regions where inequalities are graded along the socioeconomic continuum, inequalities in the uptake of colorectal cancer screening in Spain seem to be concentrated first in the most disadvantaged group and second in the least deprived group. The correlation of deprivation with FIT-positivity and faecal haemoglobin below the positivity threshold is worrying due to its association with colorectal cancer and overall mortality.

## Introduction

Colorectal cancer (CRC) is the malignancy with the highest incidence in Spain and is the second leading cause of cancer-related mortality in both sexes [1]. Screening by means of the guaiac-based faecal occult blood test (FOBT) reduces CRC-related mortality [2]. Faecal immunochemical tests (FIT) have recently been demonstrated to be more effective than guaiac-based FOBT, due to both a higher uptake and a higher detection rate of advanced neoplasia [3], and have been associated with a significant reduction in CRC incidence [4] and mortality [5].

To be effective and efficient, population-based screening programmes must achieve strong adherence, and thus high uptake rates in screening and equity in access are critical in any organised programme [6]. However, there is considerable evidence of substantial variation in uptake by age, gender, socioeconomic status and ethnicity in different regions [7–15]. Besides uptake, other screening outcomes vary depending on these variables. Faecal haemoglobin concentrations (f-Hb), and therefore positivity rates, are significantly higher in men and increase with age and greater deprivation [9,13,16,17], among other factors. The positive predictive value of FIT for neoplasia and advanced neoplasia has been reported to be higher for men and older persons, but its association with deprivation is not yet clear [9,18]. If maintained, these differences could lead to unequal mortality reductions.

The aim of this study was to describe the distribution of the main outcomes of the first round of a population-based colorectal cancer screening programme by socioeconomic level, taking into account age and sex.

## Materials and methods

### Study setting and population

The Barcelona colorectal cancer screening programme (BCRCSP) started in December 2009 and is currently running its fourth round. The programme follows the European Guidelines[6], is entirely free of charge and biennially invites men and women aged 50 to 69 years to a FIT test (OC-Sensor, Eiken Chemical Co. Ltd, Tokyo, Japan; cut-off point set at 100ng haemoglobin [Hb]/mL buffer, equivalent to 20μg Hb/g faeces). Pharmacies serve as the FIT collection point. The results of the first round of the BCRCSP and the screening process have been published in more detail elsewhere [19].

The eligible population of the first round of the BCRCSP comprised 183,187 men and women aged 50 to 69 years who were invited to participate between 12.01.2009 and 12.31.2011. People without socioeconomic data were excluded (10,225, 5.6%), yielding a final study population of 172,962 persons.

### Variables and data source

Basic sociodemographic data (age, sex and postal address) and variables related to the screening process (e.g. date of invitation, exclusion category, test result) were drawn from the BCRCSP data system. The Public Health Agency of Barcelona provided the scale known as the *Medea deprivation index*, based on the results of the Medea Project [20]. This composite deprivation index was created on the basis of five indicators from the 2001 census: unemployment, manual occupation, casual employment, insufficient education (total) and insufficient education among young people (16 to 29 years old). For the city of Barcelona, the index ranges from -1.92 (least deprived) to 4.34 (most deprived). Five groups were constructed on the basis of the census tract quintiles of the city (deprivation quintiles, Dq), where 1 corresponds to the least deprived quintile and 5 to the most deprived quintile. Each person in the study population was assigned a deprivation index value and its corresponding Dq by linking that person’s postal address with the BCRCSP database. There were no differences in the age and sex distribution of the excluded and eligible populations. The sensitivity analysis comprising excluded persons in the models did not modify any of the results.

### Definitions

*Screened (participants)*: Those who returned the test and the laboratory provided a valid result.

*Uptake*: the number of participants divided by the eligible study population (%).

*FIT positivity rate*: the number of people with a FIT-positive result divided by those screened (%).

*Colonoscopy adherence rate*: the number of persons undergoing colonoscopy divided by persons with a positive FIT result (%).

*Advanced neoplasia*: Those with a colonoscopy result indicating medium- and high-risk adenoma or cancer, expressed as a percentage of all colonoscopy results (positive predictive value [PPV] for advanced neoplasia) and as a detection rate (per 1000 participants).

### Statistical analyses

A descriptive analysis was conducted for all variables by outcomes. The adjusted effects of age, sex and deprivation on uptake, FIT positivity, colonoscopy adherence and advanced neoplasia detection rates were analysed using logistic regression models. Linear *P for trends* were tested for categorised age data and deprivation quintiles. *P values* less than 0.05 were considered statistically significant using a 2-sided test. Analyses were performed using SPSS, version 22.0.

### Ethical considerations

The study followed the principles embodied in the Declaration of Helsinki and approval was obtained from the Clinical Research Ethical Committee of the Parc de Salut Mar in July 2014. All databases were anonymised; addresses were erased after linkage to obtain the *Medea deprivation index*.

## Results

**Fig 1:**
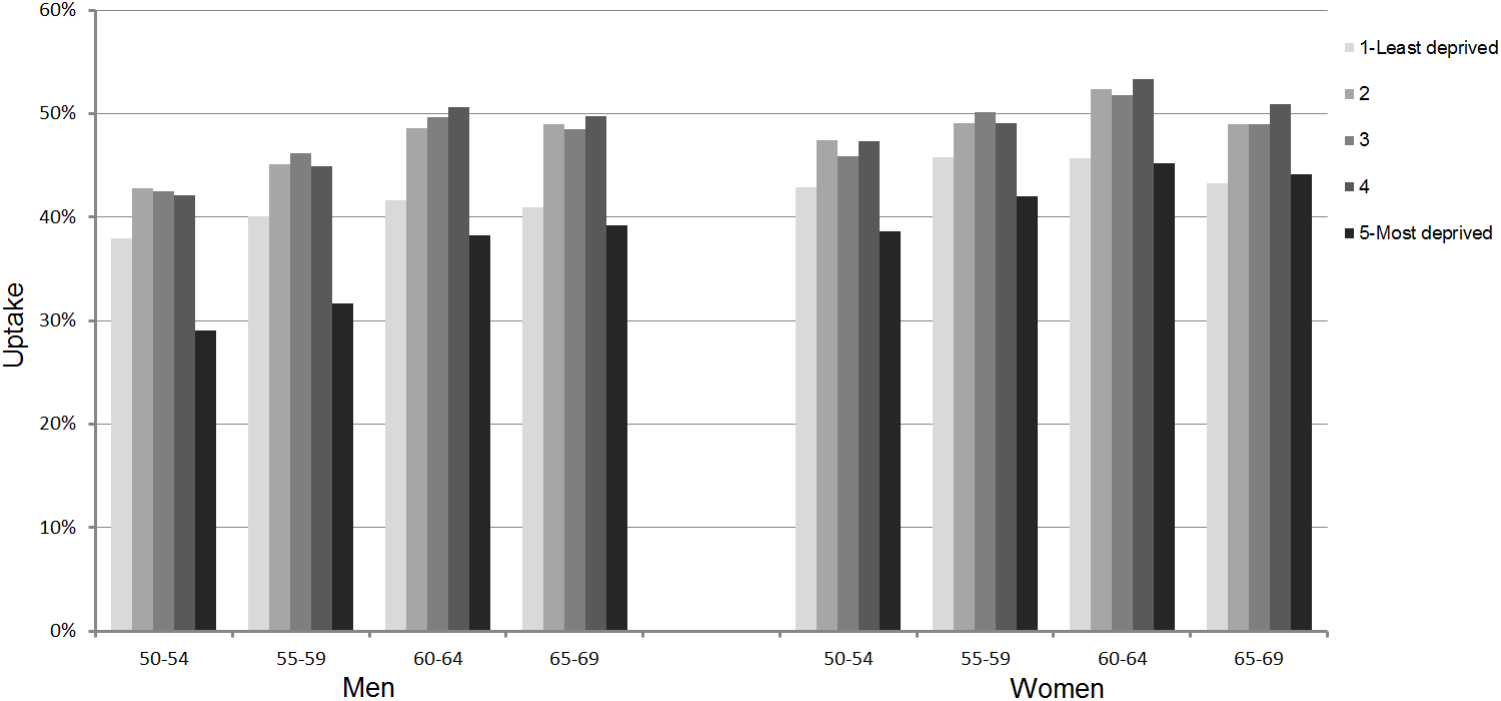
Uptake by age and deprivation in men and women.

Mean age of the eligible population was 58.3 years (SD 5.7). Overall uptake by the study population was 44.7% and was higher among women and persons aged 60–64 years (Table 1). In both sexes, uptake was lowest in the most deprived Dq (33.4% in men, 42.1% in women), followed by the least deprived Dq (38.8% in men, 44.4% in women) and was highest in the intermediate Dq. This pattern of uptake by socioeconomic level was consistent in all age groups among men and women (Fig 1). The absolute difference between the uptake rates in the most deprived group and the intermediate groups was greater in men and in the younger age categories. After adjustment by age and sex, the uptake rate was significantly lower in Dq 5 than in Dq 1 (OR 0.84, 95% CI 0.81–0.86), and the odds of participating were highest in Dqs 2, 3 and 4 (Table 2).

**Table 1:** Sociodemographic characteristics of the eligible study population, those screened, those with a faecal immunological test (FIT) positive result, those who undergo colonoscopy and those with advanced neoplasia.

	Eligible population (n = 172962)	Uptake (n = 77349)		FIT positive (n = 4764)		Colonoscopy adherence (n = 4003)		Advanced Neoplasia (High risk adenoma & cancer) (n = 1893)		
	n	n	% out of Invited	n	% out of uptakers	n	% out of FIT positives	n	Positive Predictive Value^1^	Per 1000 uptakers
**TOTAL**	172962	77349	44.72%	4764	6.16%	4003	84.03%	1893	47.29%	24.5
**Age**										
50–54	55222	23074	41.78%	1025	4.44%	855	83.41%	340	39.77%	14.7
55–59	43571	19441	44.62%	1130	5.81%	960	84.96%	455	47.40%	23.4
60–64	41228	19647	47.65%	1431	7.28%	1197	83.65%	600	50.13%	30.5
65–69	32941	15187	46.10%	1178	7.76%	991	84.13%	498	50.25%	32.8
**Deprivation**										
1-Least deprived	48045	20400	42.46%	1109	5.44%	905	81.61%	445	49.17%	21.8
2	34572	16509	47.75%	957	5.80%	808	84.43%	378	46.78%	22.9
3	29128	13863	47.59%	871	6.28%	756	86.80%	340	44.97%	24.5
4	33374	16105	48.26%	1086	6.74%	942	86.74%	463	49.15%	28.7
5-Most deprived	27843	10472	37.61%	741	7.08%	592	79.89%	267	45.10%	25.5
**MEN**										
Total	80926	34185	42.20%	2692	7.90%	2267	84.20%	1254	55.30%	36.7
**Age**										
50–54	26501	10275	38.80%	585	5.70%	495	84.60%	223	45.10%	21.7
55–59	20483	8514	41.60%	627	7.40%	531	84.70%	306	57.60%	35.9
60–64	18951	8633	45.60%	809	9.40%	675	83.40%	396	58.70%	45.9
65–69	14991	6763	45.10%	671	9.90%	566	84.40%	329	58.10%	48.6
**Deprivation**										
1-Least deprived	21432	8578	40.00%	622	7.30%	517	83.10%	287	55.50%	33.5
2	15910	7300	45.90%	531	7.30%	445	83.80%	251	56.40%	34.4
3	13600	6270	46.10%	496	7.90%	433	87.30%	229	52.90%	36.5
4	15691	7267	46.30%	636	8.80%	545	85.70%	306	56.10%	42.1
5-Most deprived	14293	4770	33.40%	407	8.50%	327	80.30%	181	55.40%	37.9
**WOMEN**										
Total	92036	43164	46.90%	2072	4.80%	1736	83.80%	639	36.80%	14.8
**Age**										
50–54	28721	12799	44.60%	440	3.40%	360	81.80%	117	32.50%	9.1
55–59	23088	10927	47.30%	503	4.60%	429	85.30%	149	34.70%	13.6
60–64	22277	11014	49.40%	622	5.60%	522	83.90%	204	39.10%	18.5
65–69	17950	8424	46.90%	507	6.00%	425	83.80%	169	39.80%	20.1
**Deprivation**										
1-Least deprived	26613	11822	44.40%	487	4.10%	388	79.70%	158	40.70%	13.4
2	18662	9209	49.30%	426	4.60%	363	85.20%	127	35.00%	13.8
3	15528	7593	48.90%	375	4.90%	323	86.10%	111	34.40%	14.6
4	17683	8838	50.00%	450	5.10%	397	88.20%	157	39.50%	17.8
5-Most deprived	13550	5702	42.10%	334	5.90%	265	79.30%	86	32.50%	15.1

^1^ PPV, Positive Predictive Value (% out of all colonoscopies)

**Table 2:** Adjusted odds ratios for uptake, FIT positivity, colonoscopy compliance and advanced neoplasia.

	Uptake (Uptakers vs. Non-uptakers; among elegible) (n = 77349)			FIT positivity (FIT positives vs. FIT negatives; among participants) (n = 4764)			Colonoscopy adherence (FIT positives who undergo colonoscopy vs. Non-adherents) (n = 4003)			Advanced neoplasia (High risk adenoma & cancer vs. All other participants) (n = 1893)		
	n	OR^1^	(95% CI)	n	OR^1^	(95% CI)	n	OR^1^	(95% CI)	n	OR^1^	(95% CI)
**Sex**												
Men	34185	1		2692	1		2267	1		1254	1	
Women	43164	1.198	(1.175–1.221)	2072	1	(0.557–0.627)	1736	1	(0.834–1.141)	639	0.395	(0.358–0.435)
**Age**												
50–54	23074	1		1025	1		855	1		340	1	
55–59	19441	1.120	(1.092–1.149)	1130	1.340	(1.229–1.462)	960	1.124	(0.891–1.418)	455	1.621	(1.406–1.868)
60–64	19647	1.264	(1.232–1.297)	1431	1.708	(1.572–1.855)	1197	1.019	(0.820–1.266)	600	2.130	(1.861–2.437)
65–69	15187	1.192	(1.159–1.225)	1178	1.815	(1.664–1.979)	991	1.074	(0.855–1.349)	498	2.273	(1.976–2.614)
*p Trend*	*<0*.*001*			*<0*.*001*			*0*.*738*			*<0*.*001*		
**Socioeconomic level**												
1-Least deprived	20400	1		1109	1		905	1		445	1	
2	16509	1.251	(1.216–1.286)	957	1.073	(0.982–1.174)	808	1.222	(0.970–1.541)	378	1.050	(0.914–1.207)
3	13863	1.250	(1.214–1.287)	871	1.175	(1.072–1.288)	756	1.482	(1.156–1.900)	340	1.131	(0.980–1.305)
4	16105	1.276	(1.240–1.312)	1086	1.241	(1.138–1.354)	942	1.478	(1.172–1.864)	463	1.298	(1.137–1.481)
5-Most deprived	10472	0.836	(0.811–0.861)	741	1.317	(1.196–1.451)	592	0.897	(0.709–1.135)	267	1.158	(0.993–1.351)
*p Trend *	*<0*.*001*			*<0*.*001*			*<0*.*001*			*0*.*002*		

^1^ Variables included in the model: sex, age, socioeconomic level.

**Fig 2:**
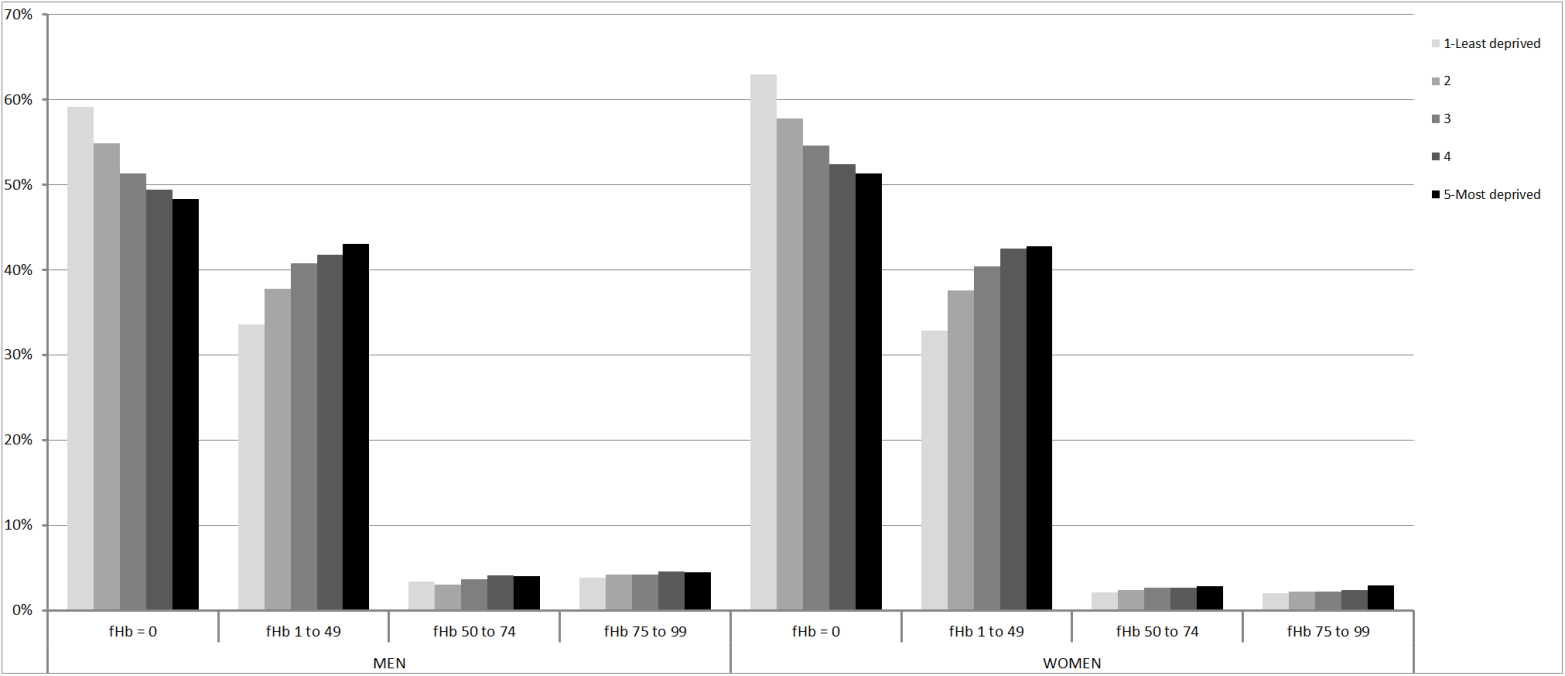
Distribution of faecal haemoglobin (f-Hb) results by deprivation and sex among participants. Percentage out of all faecal immunological test (FIT) results within each deprivation group.

A total of 4,764 people had a positive FIT result (6.2%). As expected, FIT positivity rates were higher in men than in women and increased with age (p-trend<0.001) and deprivation (p-trend<0.001), both in men (7.3% in Dq 1, 8.5% in Dq 5) and in women (4.1% and 5.9%, respectively) (Tables 1 and 2). Table 3 shows the distribution of f-Hb percentiles for all participants and for men and women by deprivation categories. Undetectable f-Hb was found in 55.2% of the participants. As expected, all percentiles were higher in men than in women. The 90%, 95% and 97.5% percentiles for f-Hb consistently increased with Dq in both sexes. The distribution of f-Hb concentration among deprivation categories is shown in Fig 2. Of note, the proportion of people with undetectable f-Hb decreased with deprivation in both sexes, whereas the proportion of people with detectable f-Hb below the threshold of 100ng Hb/mL buffer (i.e. f-Hb between 1 and 99ng Hb/mL buffer) increased with deprivation.

**Table 3:** Percentiles of faecal haemoglobin results by sex and deprivation.

	n	25.00%	50.00%	75.00%	90.00%	95.00%	97.50%
TOTAL							
1-Least deprived	20400	0.0	0.0	5.0	30.9	116.0	357.0
2	16509	0.0	0.0	5.0	35.0	127.0	435.0
3	13863	0.0	0.0	6.0	37.0	147.0	458.8
4	16105	0.0	0.0	7.0	43.0	152.0	541.4
5-Most deprived	10472	0.0	1.0	7.0	50.0	180.0	591.1
MEN							
1-Least deprived	8578	0	0	6	50	200	644.3
2	7300	0	0	7	52	220.9	714.5
3	6270	0	0	7	56	232.5	719.7
4	7267	0	1	9	78.2	250.4	897
5-Most deprived	4770	0	1	9	78	250	817.7
WOMEN							
1-Least deprived	11822	0	0	4	21	72	224.4
2	9209	0	0	5	25	89.5	243.7
3	7593	0	0	5	25	98	249
4	8838	0	0	6	27	102	289
5-Most deprived	5702	0	0	7	36	124	376.8

Overall colonoscopy adherence was 84.0% and was higher in Dq 3 and 4, with no significant differences for the remaining categories or variables in the adjusted models (Tables 1 and 3). Advanced neoplasia was diagnosed in 1,893 people (PPV 47.3% out of all colonoscopies), yielding an overall detection rate of 24.5 per 1000 participants. The PPV, detection rates, and odds of being diagnosed with an advanced neoplasia were significantly higher in men and increased with age. PPV was lowest for women in Dq 5 and, after adjustment, those in Dq 4 had a statistically significant higher odds of being diagnosed with advanced neoplasia.

## Discussion

The results of this study show that socioeconomic deprivation has a significant impact at all stages of the FIT-based colorectal cancer screening pathway. Inequalities in the uptake of CRC screening seem to be concentrated first in the most disadvantaged group and second in the least deprived group. Groups with the greatest deprivation have also the lowest uptake rates, highest positivity rates and lowest detection rates.

### Uptake

Uptake was lowest in the most deprived quintile, followed by the least deprived one, and was highest in the intermediate groups. Unlike our results, most studies found a linear reverse association between uptake and deprivation, i.e. the higher the deprivation level the lower the uptake [7–13,15,21]. When interpreting these results, it is important to consider the invitation strategy. In a study performed in Italy, where FOBT test kits are also distributed by pharmacies, high educational attainment and working in a non-manual occupation were positively associated with uptake [21]. In countries where tests are sent by surface mail, such as England, Scotland, Australia and more recently Denmark, uptake consistently decreases with increasing socioeconomic deprivation [7,10,11,18]. Discrepant results have been reported from countries where screening is based on the primary care network: in the Basque Country in Spain, lower uptake rates were found for the most and least deprived groups [14]; in Canada, a correlation was described between higher education and lower uptake [22], and in France the association between uptake and deprivation was contradictory [15]. In the light of these results, there is no apparent association between invitation strategy and patterns of uptake and deprivation.

A worrisome finding was that men in the most deprived groups had the lowest participation rates for FOBT screening, since the incidence of CRC has been shown also to be higher in this group [1,23]. The reasons described for non-participation among the most deprived groups are a lack of information, prioritisation of other problems, not understanding the written information provided, underestimation of the benefits of screening and stronger fears and fatalistic attitudes [24,25]. While insurance status has been described in other contexts to influence CRC screening uptake [15], in Spain health care access is guaranteed for all residents regardless of their socioeconomic level and the BCRCSP is completely free of charge throughout the whole process including all screening and diagnostic tests as well as cancer treatment. Hence, lack of private health insurance among the most deprived is not expected to play a role in the present setting.

Lower screening uptake rates in men have been related to their lesser interest in their health as well as their fear of the diagnostic test, while women may assume the role of caregiver and worry more about their health for the sake of those around them [8,14,26]. On the other hand, the low uptake found in this study among the least deprived persons might be due to their greater access to private healthcare [27], which may offer colonoscopy as an opportunistic screening test [14,22], but this possibility requires confirmation. Therefore, interventions to increase CRC awareness and prevention should, at least initially, target both the most and the least deprived groups.

### FIT results

As in previous studies [9,14,16,18], in our study male gender, increasing age and lower socioeconomic status were significantly associated with higher FOBT positivity and f-Hb concentrations. Besides differences in the prevalence of neoplastic lesions, other factors possibly related to these findings are differences in the haemoglobin blood concentration, diet, intestinal transit time and other concomitant comorbidities [9,28]. The sex-independent relationship between deprivation and f-Hb concentrations points towards non–gender-related mechanisms [16] and could be associated with the relationship between deprivation and colorectal cancer incidence, particularly in men [23,29]. Of note, among persons testing negative, the proportion of individuals with detectable f-Hb below the positivity threshold increased with deprivation. To the best of our knowledge, this correlation has not been previously reported *per se* and is of particular interest because even small values of f-HB have been related not only with CRC-specific mortality but also with overall mortality, therefore suggesting f-HB as a possible marker for mortality [30].

### Colonoscopy adherence

Colonoscopy adherence was lower among people in the most deprived groups. A negative association between deprivation and colonoscopy uptake after a positive FOBT has been reported in Scotland [10] and England [9,31], but other authors have found no correlation [11,14]. The lower adherence among the most deprived groups is worrying because of their higher CRC incidence and more restricted access to alternative sources of healthcare. Overall health is a facet of deprivation and hence more deprived individuals may be less likely to be as fit to undergo a colonoscopy than less deprived individuals [9]. Colonoscopy uptake was also lowest among the least deprived women (less so among men), which could also be related to greater access to private colonoscopy [27].

### Colonoscopy outcomes

Individuals belonging to the fourth most deprived group had a slightly increased risk of being diagnosed with advanced neoplasia. However, caution must be exercised when interpreting these results, since the sample size is small and the results are constrained both by test positivity and by colonoscopy adherence. PPV values were significantly higher for men, an observation which has already been described before and is probably related to a higher burden of disease [9,10]. Furthermore, we found a lower PPV among the most deprived women, which could point towards a higher false positive rate or lower colonoscopy adherence among deprived women with advanced neoplasia. Other authors have found an association between a higher PPV and higher cancer detection rates with increasing deprivation in both sexes and across all age groups [9].

### Strengths and limitations

This study has a large sample size including the eligible population of the first round of an ongoing population-based CRC screening programme. The use of information collected directly from the BCRCSP database ensured a high reliability of the data, avoiding the common selection bias observed in designs using self-administered questionnaires to assess screening and socioeconomic status. Moreover, the present study was able to examine in detail the different outcomes of the programme and, unlike many publications, to report differences in f-Hb concentrations by age, sex and deprivation level.

Nevertheless, the study also has several limitations. The use of an ecological deprivation index to assign individual socioeconomic level may lead to misclassification in some cases. However, many studies rely on similar indicators calculated in small areas [12,16]. Furthermore, studies using individual socioeconomic measures have obtained very similar associations between deprivation and uptake [7]. Another limitation is the time lag between calculation of the deprivation index (2001) and the start of the first round of the programme (2009). Census data in Spain is gathered every 10 years and, unfortunately, data from 2011 could not be used because from that year onwards the census in Spain ceased to be universal and started to be calculated on the basis of a representative 12% sample [32], hindering the use of indicators in small geographical areas, as in this study.

We had no access to information on ethnicity or race, country of origin or nationality. Previous studies have found that ethnic differences were almost completely explained by differences in socioeconomic factors [7]. In the city of Barcelona, many–but not all–of the neighbourhoods classified as being of low socioeconomic level also have the highest share of immigrants [33]. Lastly, the present study was not able to assess the reasons for non-participation or the personal history and colorectal outcomes of persons choosing not to participate and those testing negative.

## Conclusions

In Spain, inequalities in the uptake of CRC screening seem to be concentrated first in the most disadvantaged group and second in the least deprived group. Efforts should be made to ensure that equal access is ensured throughout the screening process, minimising inequalities in both uptake and colonoscopy adherence. The relationship between detectable f-Hb and deprivation throughout the spectrum of persons with a negative result is worrying because of its relationship with overall mortality and warrants further investigations.

Our results add to the evidence that there are substantial socioeconomic inequalities, affecting not only uptake and compliance with the diagnostic test, but also test performance. If these differences remain, some groups may derive a disproportionately lower share of the survival benefits of screening, which would inevitably increase the relative socioeconomic inequalities in the incidence of CRC and its associated mortality.
